# The Use of GLP1R Agonists for the Treatment of Type 2 Diabetes in Kidney Transplant Recipients

**DOI:** 10.1097/TXD.0000000000000971

**Published:** 2020-01-13

**Authors:** Aleksandra Kukla, Jennifer Hill, Massini Merzkani, Andrew Bentall, Elizabeth C. Lorenz, Walter D. Park, Matthew D’Costa, Yogish C. Kudva, Mark D. Stegall, Pankaj Shah

**Affiliations:** 1 Division of Nephrology and Hypertension, Mayo Clinic, Rochester, MN.; 2 Department of Surgery, Mayo Clinic, Rochester, MN.; 3 Division of Endocrinology, Diabetes, Metabolism, and Nutrition, Mayo Clinic Rochester, MN.

## Abstract

Glucagon-like peptide-1 receptor agonists (GLP1RA) have been shown to improve glucose control and diabetes-related comorbidities in patients without solid organ transplants. The effectiveness, safety, and tolerability of GLP1RA after kidney transplantation have not been adequately studied.

**Methods.:**

We retrospectively reviewed data on kidney transplant recipients performed in our institution, who were initiated on GLP1RA either for the treatment of type 2 diabetes diagnosed before transplantation or posttransplant diabetes. We analyzed efficacy, safety, and the effect on kidney allograft function.

**Results.:**

Seventeen kidney transplant recipients were initiated on GLP1RA therapy, 14 of which remained on the medication for at least 12 months. The use of GLP1RA had no significant impact on weight loss, but was associated with a significant reduction in the total daily insulin dose, from the median of 63 [interquartile range 43-113] IU to 44 [interquartile range 25-88] and reduction in the risk of hypoglycemia in patients who were on therapy for at least approximately 12 months. Kidney function remained stable and none of the recipients experienced acute rejection. Tacrolimus dose was not significantly changed. Five patients (29%) discontinued GLP1RA therapy—4 due to side effects and 1 due to uncontrolled hyperglycemia.

**Conclusions.:**

GLP1RA may be a relatively safe and effective treatment for kidney transplant recipients with type 2 diabetes that allows for a reduction in insulin requirements. More studies are needed to determine whether the use of these agents will translate into an improvement in allograft and patient survival.

Diabetes continues to be a significant burden for kidney transplant recipients as close to 40% of all patients undergoing kidney transplantation carry the diagnosis of diabetes, predominantly T2DM, and an additional 10%–20% of nondiabetic recipients develop posttransplant diabetes (PTDM).^1,2^ Both, pre-existing T2DM and PTDM adversely affect patient and kidney allograft outcomes.^2,3^ These findings obviate the need for risk factor modification in this patient population; however, data remain limited as transplant recipients are routinely excluded from clinical trials testing the novel therapies.^4,5^

Glucagon-like peptide-1 receptor agonists (GLP1RA) have emerged as an effective agent to reduce the risk of complications associated with T2DM in nontransplant recipients. Endogenous GLP1 potentiate glucose-dependent insulin secretion and inhibit glucagon secretion in pancreatic islet cells, minimizing the risk of hyoglycemia. GLP1RA bind and activate GLP-1 receptors, mimicking the action of the naturally secreted GLP-1. Downstream actions include improvement in insulin sensitivity, delay in gastric emptying, appetite suppression, and anti-atherogenic effects.^6,7^ GLP1RA differ in chemical structures and pharmacokinetic profiles and are categorized as short acting (eg, exenatide twice daily formulation and half-life 2–4 h) and long acting (eg, liraglutide and half-life 13 h). Although most of the benefits of GLP-1 can be exerted by both long-acting and short-acting formulations, short-acting agents have greater effect on gastric emptying and postprandial hyperglycemia, while long-acting GLP1RA predominantly lower fasting blood glucose.^6,8^ Benefits of GLP1RA have translated to improved cardiovascular and renal outcomes in clinical trials. Recently published meta-analysis of large randomized controlled trials showed relative risk reduction of myocardial infarction, stroke, and cardiovascular death in patients with established atherosclerotic cardiovascular disease treated with GLP1RA.^9^ Both, LEADER (liraglutide) and SUSTAIN6 (semaglutide) trials showed improvement in albuminuria,^10,11^ while the AWARD-7 trial was the first to show that dulaglutide attenuates estimated glomerular filtration rate (eGFR) decline compared with insulin glargine in patients with T2DM and moderate-to-severe chronic kidney Disease at 52 weeks follow up.^12^

Yet, there remains concern over the safety and tolerability of these agents in the kidney transplant population. This apprehension is largely centered around potential gastrointestinal (GI) side effects related to delayed gastric emptying, and, therefore possible interference with absorption of transplant medications.^2,13^ Questions regarding these issues are met with uncertainty as the current literature regarding the use of GLP1RA after kidney transplantation is limited.^14-16^ To bridge the gap of knowledge in the effects of these agents in the transplant patient population, the goal of this article is to describe our experience using GLP1RA in kidney transplant recipients with preexisting T2DM and PTDM at our center.

## MATERIALS AND METHODS

### Study Population and Patient Characteristics

The study was approved by the Institutional Review Board at Mayo Clinic, Rochester, Minnesota (MCR). This was a retrospective cohort study in which we reviewed the electronic medical records (EMR) of adult (age ≥ 18 y old) solitary or combined kidney transplant recipients that were transplanted at our center (MCR) and were started on GLP1RA over the past decade, from January 1, 2009 to May 21, 2019. Transplant recipients were included if they were diagnosed with either pretransplant T2DM or PTDM and had at least 1 follow-up visit at our center after GLP1RA initiation. Diagnosis of pre-existing T2DM was made if patients were on antidiabetic therapy before transplant. PTDM was diagnosed based on elevated fasting blood glucose ≥ 126 mg/dL, or Hb A1C ≥ 6.5% after the first 4 months posttransplant, according to ADA recommendation.^17,18^ Data of interest were manually abstracted from the EMR for assessment.

Baseline recipient demographics at the time of transplantation included patient age, sex, cause of end-stage renal disease (ESRD), weight (kg) and body mass index (BMI) (kg/m^2^), transplant type (kidney transplant alone or combined transplant), donor type and maintenance immunosuppression. All recipients were started on the long-acting GLP1RA. Diabetic parameters included in the analysis were type of diabetes (preexisting T2DM versus PTDM), fasting blood glucose, hemoglobin A1C (HbA1C) as well as prescribed treatments in addition to GLPR1As. Patient demographics assessed at the time of GLP1RA treatment initiation were weight, BMI, creatinine with eGFR, hemoglobin, HbA1C, total fasting cholesterol level, 24-hour urine protein, and total insulin dose (if used).

### GLP1RA Treatment and Outcomes

Collected information regarding GLP1RA therapy included the type and dose of GLP1RA, along with time from transplantation to initiation of GLP1RA, reasons for initiation, other antidiabetic therapy and total daily dose of insulin, if any, at the time of GLP1RA therapy implementation and a history of hypoglycemia.

The safety and tolerability of GLP1RA was assessed in all recipients and included side effects of GLP1RA reported at the time of follow-up visit and reasons for discontinuation of GLP1RA therapy, adjustments to immunosuppression (within 4 mo of therapy initiation), and history of rejections.

Next, we identified the patients who were on therapy for at least approximately 12 months. The outcomes of interest assessed in this group included change in creatinine, renal function (eGFR in mL/min/1.73 m2 as estimated by modification of diet in renal disease study equation),^19^ weight, BMI, hemoglobin, HbA1C, reported frequency and severity of hypoglycemic symptoms, and total daily insulin dose (IU) at the time of therapy initiation and at 12 months on treatment.

Lastly, we identified recipients who were on therapy for longer than 12 months and assessed the individual change in weight and kidney function between the therapy initiation and the last follow up.

### Statistical Analysis

Statistical analysis was performed using JMP software, version 14.^20^ Statistical descriptions, including N (%) or median [interquartile range (IQR)] were used to characterize eligible participants. Patients who were on treatment for at least approximately 12 months were included in the further analysis. The clinical and laboratory outcomes were compared by the Wilcoxon Signed Rank test in the paired analysis of the data at the time of medication initiation and approximately 12 months on therapy and at last follow up or medication discontinuation if longer than 12 months. A *P*-value of <0.05 was considered to be statistically significant.

## RESULTS

### Patient Characteristics

Seventeen kidney transplant recipients were initiated on GLP1RA during the study period. Baseline characteristics of recipients at the time of kidney transplant are presented in Table 1. Most patients were male, 14 (82%) received kidney transplant alone (KTA), 1 (5.9%) received combined liver-kidney transplant, and 2 (11.8%) received combined heart–kidney transplant. Median weight was 101.7 [IQR: 90.4-115.4] kg and BMI was 34.1 kg/m^2^ [IQR: 29.5-36.1]. The most common cause of ESRD was autosomal dominant polycystic kidney disease. Maintenance immunosuppression consisted mainly of tacrolimus (16/17 patients, 94%) with the median daily dose of 5 [IQR 3-6] mg daily, while 1 recipient was on everolimus. Mycophenolate mofetil was used in 13/17 (78%) and mycophenolate acid in 2/17 (12%). Prednisone was used in 11/17 (65%) of recipients, with majority patients (10) taking 5 mg and 1 patient taking 10 mg daily.

**TABLE 1.**
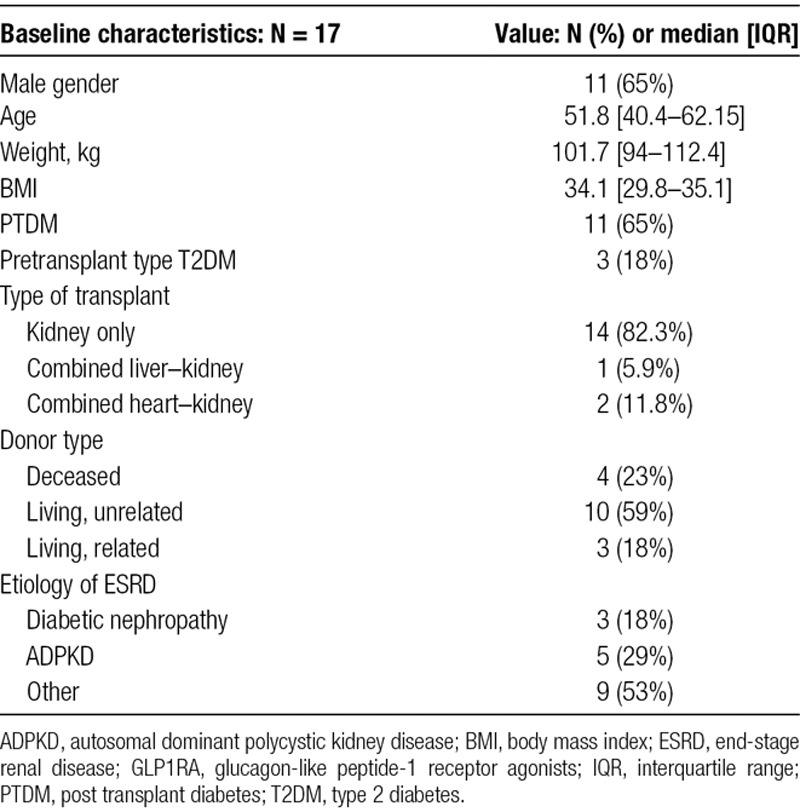
Baseline characteristics of kidney transplant recipients treated with GLP1RA

The main reported reasons for initiation of GLP1RA were to promote weight loss, improve blood glucose control, or decrease insulin requirements. Eight out of 17 patients (47%) reported mild to moderate hypoglycemic symptoms 1–3 times per week. Median time from the kidney transplant to the initiation of GLP1RA was 3.9 [IQR 1.0–9.9] years. Detailed information regarding GLP1RA type, dose, and the other antidiabetic medications is presented in Table 2. All, but 24 recipients were already on other pharmacotherapy for diabetes, with the insulin being the most commonly used agent, either alone (6 patients, 35%), in combination with metformin (5 patients, 29%), or glipizide and pioglitazone (1 patient, 6%). Majority of patients (14; 82%) were started on liraglutide.

**TABLE 2.**
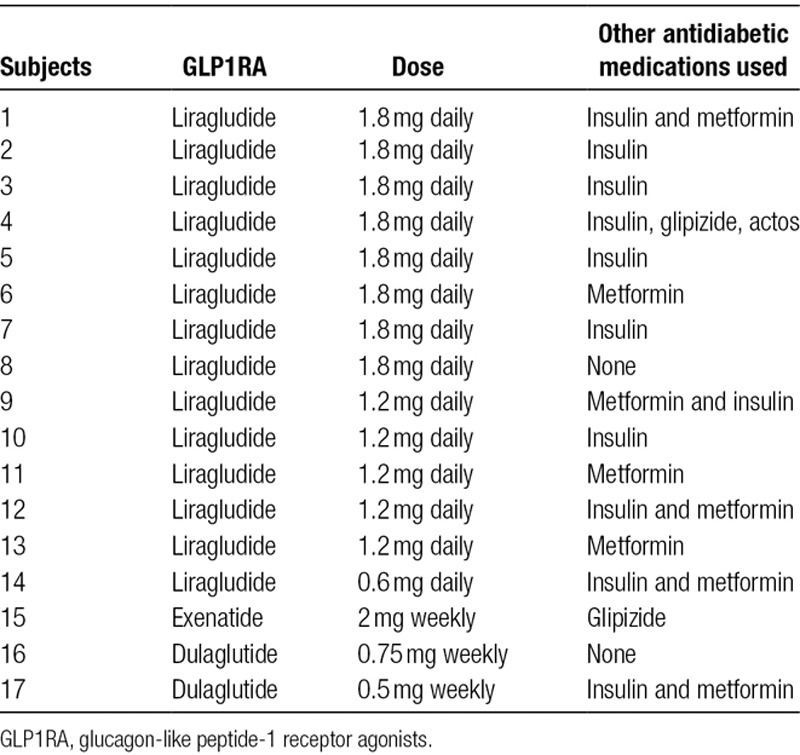
Coadministration of GLP1RA with the other antidiabetic medications

#### Outcomes After Initiation of GLP1RA Therapy

Two recipients discontinued GLP1RA within the first 2 months and were excluded from the further analysis. Detailed information regarding side effects and the reasons for discontinuation are presented in Table 3 and described in the section below. Additionally, 1 recipient underwent weight loss procedure (intragastric balloon placement) and was also excluded as weight loss achieved through the bariatric procedure could have interfered with the data interpretation. A total of 14 kidney transplant recipients underwent further outcome analysis.

**TABLE 3.**
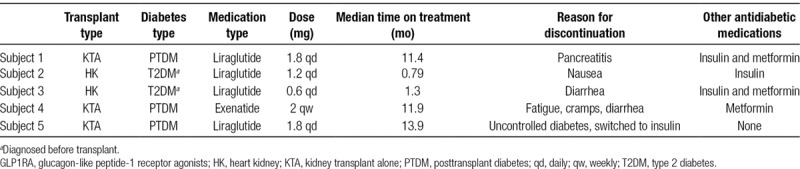
Reasons for discontinuation of GLP1RA

**FIGURE 1.**
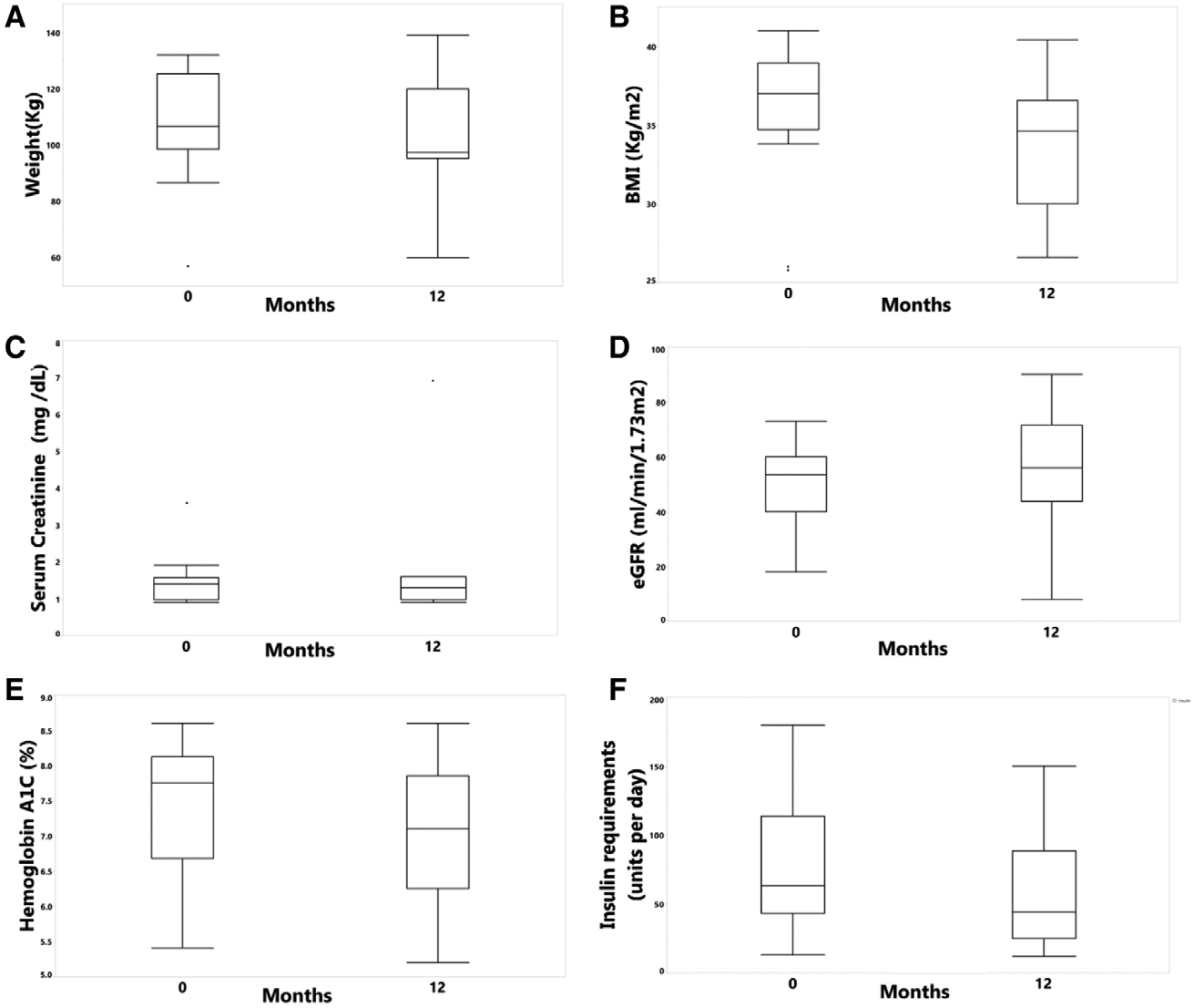
Boxplot and scatterplot. Change of parameter from the initiation of GLP1RA to 12 mo on therapy: (A) weight (kg), (B) body mass index (kg/m^2^), (C) serum creatinine (mg/dl), (D) eGFR (ml/min/1.73 m2), (E) hemoglobin A1C (%), and (F) insulin requirements (IU per d). BMI, body mass index; eGFR, estimated glomerular filtration rate; GLP1RA, glucagon-like peptide-1 receptor agonists; IU, international units.

Table 4 and Figure 1 depict the outcomes of recipients at approximately 12 months of the therapy initiation (median 12.0 [IOR 11.7-12.9] mo). Median body weight and body mass index at the therapy implementation was 106 [IQR 98.5-125.2] kg and 36.5 [IQR 34.7-38.1] kg/m^2^, respectively, and was not statistically different at approximately 12 months of follow up. While fasting blood glucose and HbA1C remained statistically unchanged (*P* = 0.07), there was a substantial reduction in total daily dose of insulin by the median of 30 IU [–36 to 2.5] in the entire cohort (*P* = 0.007). Six out of 14 recipients (43%) reported mild to moderate symptoms of hypoglycemia (1–3 times per wk) at the time of medication initiation. GLP1RA therapy allowed for substantial reduction of insulin dose by the median 34 IU in this subset of recipients [IQR 30.75-36.5] while HbA1C was at target (median of 7.0 [IQR 6.6-7.7]) at 12-month follow-up visit. Patients reported resolution (4 patients) or marked improvement in severity (mild) and frequency (1–2 per mo) of hypoglycemic events (2 patients).

**TABLE 4.**
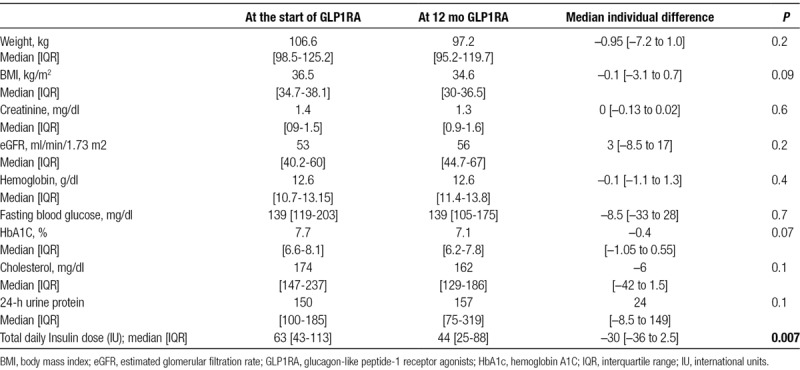
Clinical and laboratory parameters of 14 kidney transplant recipients who were on GLP1RA for at least approximately 12 mo

Median eGFR at therapy initiation was 52 [IQR 40-60] mL/min/1.73 m2 and kidney function remained stable. Tacrolimus dose did not require adjustments and was not significantly changed within 4 months of the initiation of therapy (*P* = 0.3). Total serum cholesterol and 24 urine protein excretion remained statistically unchanged (Table 4).

There were 7 recipients with follow-up longer than 12 months on GLP1RA therapy with treatment duration of 24.9 [16.1-54.4] months. Weight loss from the therapy initiation to last follow-up was 8.6 [IQR 1.2-12.5] kg (*P* = 0.07). Kidney function was not significantly changed and recipients did not report any side effects during the longer follow up. None of the recipients experienced acute rejection after medication initiation.

#### Tolerability of GLP1RA

Five patients (29%) discontinued GLP1RA—4 due to side effects and 1 was switched by primary care provider to insulin due to uncontrolled diabetes. Detailed description of patients who discontinued GLP1RA is presented in Table 3. One patient developed clinical acute pancreatitis, which resolved after the drug discontinuation. CT of abdomen/pelvis performed during that episode failed to demonstrate any mechanical cause of pancreatitis. Three other recipients developed significant GI side effects, including nausea and diarrhea. Symptoms resolved after the drug discontinuation in all patients. Kidney function remained stable in all 4 recipients who discontinued the medication due to GI side effects (individual change from the therapy initiation to drug discontinuation of 0.2 mg/dL). One recipient subsequently restarted GLP1RA, 8 months after discontinuation, and has not reported significant side effects at current 3 months of follow up.

## DISCUSSION

Our results show that therapy with GLP1RA has no adverse effect on the kidney allograft outcomes, has a similar side effects profile to the general population, and has no significant impact on Tacrolimus dosing. While these agents are associated with weight loss, we did not show a statistically significant change in our patients at 12 months of therapy initiation. Those who were on GLP1A for longer than 12 months had greater, albeit not statistically significant, weight loss likely due to the small number of recipients. Importantly, GLP1RA therapy facilitated significant insulin dose reduction, altogether leading to resolution or at least marked improvement in severity and frequency of previously reported hypoglycemic events while diabetes remained well controlled.

GI side effects, leading to the drug discontinuation, affected 4 (23%) of our patients, incidence similar to the nontransplant population.^21^ Whether or not combining GLP1RA with metformin, which may have a similar impact on GI tract,^22^ exacerbates these symptoms is unknown but may be worth further exploration. Furthermore, immunosuppressive medications (particular mycophenolate mofetil) are known to cause GI side effects, which may have a cumulative effect with GLP1RA in transplant recipients. We do not recommend mycophenolate mofetil dose reduction or discontinuation in those with GI symptoms since alteration of the dose has been previously shown to negatively impact allograft outcomes.^23^ Appropriate counseling prior the initiation of GLP1RA regarding side effects and gradual dose escalation may minimize side effects and prevent premature discontinuation of the drug in the appropriate clinical setting. One recipient developed pancreatitis, which was previously described on GLP1RA therapy, but overall is considered very rare, with recent meta-analysis of randomized controlled trials failing to show any significant increase in risk.^24^ Therefore, it is difficult to attribute pancreatitis to GLPRA use in 1 patient; however, it is a concerning potential adverse effect that requires further consideration.

Improvement in metabolic outcomes of recipients of kidney transplant remains a major unmet need in kidney transplantation.^25^ Traditionally, the focus of the transplant community has been on prevention or mitigation of immunologic graft injury or immunosuppression related side effects, with limited emphasis placed on posttransplant metabolic complications. However, there is an increasing prevalence of weight gain, obesity, and metabolic syndrome, and thus there is a growing need to appropriately manage these conditions in transplant recipients.^26-28^ This unfortunate trend was also reflected in our series of recipients, who gained weight in the lead time between the transplant and medication initiation. Obesity and weight gain has negative impact on graft survival and is at least partially responsible for diabetes, which supports more focused, weight-centered approach to treatment.^28-30^ Although we were not able to show significant weight loss in our patients, the trend was a reduction in weight and significant decrease in insulin dose, which may have further beneficial effects on both weight loss and amelioration of hypoglycemic events.

GLP1RA target key pathophysiologic pathways of micro-and macro-vascular complications with benefits extending beyond glucose control. The question remains whether these novel therapeutic agents should be routinely recommended in transplant recipients with PTDM and pre-existing diabetes given the current limitations of the literature in this area. Large studies with defined cardiac and renal endpoints are unlikely to be replicated in a transplant population, given the challenges in study design to adequately demonstrate the benefits of medication in the relatively small number of transplant recipients with largely heterogeneous diabetes phenotypes, who carry pre-existing comorbidities, unequal dialysis vintage, and superimposed transplant-related cardiovascular and renal risks in the relatively short-term study frame. However, surrogate endpoints, previously demonstrated in nontransplant populations, including improvement in weight, lipid profile, natriuresis, albuminuria, and inflammatory markers could be further explored.^31^ Moreover, histologic changes of diabetic nephropathy could constitute another important surrogate endpoint. Our group has previously shown that biopsy proven diabetic nephropathy, as manifested by moderate-to-severe mesangial expansion, affects approximately half of the recipients with diabetes (52.0% of patients with baseline diabetes and 47.1% of patients with PTDM).^32^ Others showed that these changes can develop irrespectively of glucose control.^33^ Our current and previously reported case series,^14–16^ which seem to demonstrate relative safety and tolerability of GLP1RA, may serve as preliminary results for future intervention trials on the larger number of kidney transplant recipients with well-defined clinical and histologic endpoints.

Current treatment strategies of transplant recipients with diabetes are limited and relate only to patients with PTDM.^34^ No particular agent is recommended, citing the lack of head to head comparison trials. Moreover, the guidelines do not address the needs of the patients with pre-existing T2DM at transplant. The presence of advanced renal failure before transplant significantly limits the options of oral antidiabetic medications, due to altered pharmacokinetics of the drugs, and many transplant candidates with type 2 diabetes are on insulin.^35^ Others may be able to come off glucose lowering agents as endogenous insulin clearance decreases with the progressive renal disease.^36^ In the immediate posttransplant period, insulin is recommended for blood glucose control, due to the instability of kidney function and immunosuppressive medications.^18^ At 3–4 months posttransplant, treatment of T2DM should be routinely re-evaluated for addition of oral medications, which can provide benefits beyond the glucose control. Adding another agent, like GLPRA may also improve treatment burden through the reduction in the insulin requirements, therefore potentially improve patient-centered outcomes, but that remains to be studied.^37,38^ Altogether, it is important to avoid clinical inertia in the treatment of diabetes in kidney transplant recipients.

A wide array of oral pharmacotherapy for diabetes is available and endorsed by ADA. Metformin is considered a first choice in general population with T2DM, based on the UK Prospective Diabetes Study (UKPDS), which showed cardiovascular benefits in overweight type 2 diabetes patients; however, these results were not confirmed in the more recent era.^39,40^ In kidney transplant recipients, metformin has not been adequately studied, and there are safety concerns in the setting of decreased kidney function.^41^ In patients with established atherosclerotic cardiovascular disease, ADA recommends GLP1RA as a second line of therapy, which may be also beneficial in those with obesity and compelling need to prevent hypoglycemia.^42^ While alternative agents are also recommended in specific clinical scenarios, detailed recommendations are beyond the scope of this article and are in the official ADA statement.^42^ Here, we describe our overall positive experience with GLP1RAs, which lead us to believe that these agents should be more commonly used in transplant recipients, with both, PTDM and preexisting T2DM, especially if specific comorbidities are present as endorsed by ADA.^42^

Limitations of our study include retrospective nature and small number of recipients. However, this study represents a real life scenario and supports the further exploration of benefits of GLP1RA in kidney transplant population. Assessment of Tacrolimus levels was done with 12-hour troughs, as we do not routinely measure Tacrolimus area under the curve (AUC), often used often used as the gold standard to assess Tacrolimus absorption. However, clinical studies have shown a good correlation between AUC and trough concentration (determined at 12-h postdose),^43^ which is monitored in our institution. After the initiation of GLP1RA, no dose changes were required and none of the patient experienced acute rejection; therefore, we believe that there was no clinically significant influence of GLP1RA on tacrolimus absorption.

## CONCLUSION

GLP1RAs may be a safe option for kidney transplant recipients. More studies are needed to solidify their role in the management of kidney transplant recipients with diabetes and the optimal timing of the therapy initiation. The question remains whether or not GLPRA translates into improved patient and allograft, and this requires further exploration.
